# The Reliability and Validity of Active Range of Motion Measurement for Fingers Using Three-Dimensional Motion Analysis

**DOI:** 10.3390/s26196162

**Published:** 2026-09-29

**Authors:** Saki Moriguchi, Takashi Takebayashi, Jumpei Arata, D. S. V. Bandara, Ruiko Matsumoto, Shinichi Shimada, Nobutaka Mukae

**Affiliations:** 1 Department of Rehabilitation, Itami Kousei Neurosurgical Hospital, Itami 664-0028, Japan; 2 Graduate School of Rehabilitation Science, Osaka Metropolitan University, Osaka 536-8525, Japan; takshi77@omu.ac.jp; 3 Department of Mechanical Engineering, Faculty of Engineering, Kyushu University, Fukuoka 819-0395, Japan; jumpei@mech.kyushu-u.ac.jp (J.A.); d-sanjaya@mech.kyushu-u.ac.jp (D.S.V.B.); 4 Department of Physical Medicine and Rehabilitation, Itami Kousei Neurosurgical Hospital, Itami 664-0028, Japan; matsumoto_r@itami-kousei.jp (R.M.); shimadashin1024@ybb.ne.jp (S.S.); 5 Department of Neurosurgery, Itami Kousei Neurosurgical Hospital, Itami 664-0028, Japan; 6 Department of Neurosurgery, Graduate School of Medical Sciences, Kyushu University, Fukuoka 812-8582, Japan; mukae.nobutaka.882@m.kyushu-u.ac.jp

**Keywords:** stroke, range of motion, finger function, reliability, validity

## Abstract

The Fugl–Meyer Assessment (FMA) and Range of Motion (ROM) are well-known measures for evaluating finger motor function. However, the finger FMA has a narrow scoring range, and goniometer ROM measurements lack reliability. Therefore, we investigated ROM measurement using a 3D motion capture system (Ultraleap Stereo IR 170, Ultraleap Ltd., Bristol, UK, hereinafter referred to as Ultraleap). This study aimed to evaluate the intra- and inter-rater reliability of finger Active ROM (A-ROM) using Ultraleap, and its criterion-related validity compared to a goniometer, in stroke patients with upper limb hemiplegia. A-ROM was evaluated over a 3-day period. For Ultraleap reliability, flexion intraclass correlation coefficients (ICCs) showed variability (intra-rater: 0.576–0.896; inter-rater: 0.426–0.841), whereas extension ICCs were ≥0.81 for all joints except the thumb interphalangeal (IP) joint. Regarding criterion-related validity, correlation coefficients ranged from −0.0706 to 0.493 for flexion, and 0.698 to 0.814 for extension (excluding the thumb IP joint). Ultraleap extension measurements demonstrated acceptable-to-high reliability and moderate-to-strong correlations with manual goniometry in most joints, except for the thumb IP joint. However, Bland–Altman analyses revealed systematic and proportional biases, indicating limited agreement for absolute ROM values. While the system cannot be directly interchangeable with manual goniometry for absolute ROM assessment, it may serve as an efficient supplementary tool for repeated measurements of finger extension in clinical settings.

## 1. Introduction

The Fugl–Meyer Assessment (FMA) and Range of Motion (ROM) are well-established, representative indices for evaluating finger motor function. However, the finger subscale of the FMA has a narrow scoring range and uses a 3-point ordinal scale for each item, making it insensitive to subtle changes in motor function. Additionally, while goniometers are standard tools for measuring finger ROM, they lack sufficient reliability [1], which poses a challenge for ensuring consistency, particularly in multicenter clinical trials. Furthermore, the goniometric method allows for the measurement of only one joint at a time; consequently, evaluating all finger joints is time-consuming and places a substantial burden on both patients and therapists.

To address these limitations, markerless motion capture devices have attracted attention as cost-effective and user-friendly alternatives. Several studies have investigated finger ROM measurements using the Leap Motion Controller (Ultraleap Ltd., Bristol, UK; formerly Leap Motion, San Francisco, CA, USA) [2,3]. However, these investigations were restricted to healthy individuals, and to our knowledge, no studies have evaluated stroke patients. Although Nizamis et al. [3] highlighted the clinical potential of the Leap Motion Controller, they emphasized the need to verify its applicability in subjects with impaired finger function. Therefore, investigating the reliability and validity of detecting subtle functional changes in the paretic hand of clinical populations is crucial. Recently, a successor device with improved accuracy, the Ultraleap Stereo IR 170 (Ultraleap Ltd., Bristol, UK, hereinafter referred to as Ultraleap), has become available. Furthermore, a recent systematic review highlighted the rapidly growing clinical application of markerless motion capture (MMC) technology in physical assessment and rehabilitation [4]. Although recent studies have evaluated the accuracy and tracking performance of modern video-based optical sensors during dynamic hand and finger tasks in individuals with upper-body impairments [5], comprehensive validation regarding multi-joint finger active ROM—especially across individual joints and movement directions in post-stroke patients—remains insufficient.

Thus, this study aimed to examine the intra- and inter-rater reliability of finger active range of motion (A-ROM) measurements using the Ultraleap Stereo IR 170 and to examine their concurrent association and agreement with conventional goniometry in patients with post-stroke upper-limb hemiplegia. Establishing the measurement properties of this digital method may support its use as a time-efficient supplementary approach for finger A-ROM assessment in clinical practice. Its responsiveness to longitudinal clinical change requires further prospective investigation.

## 2. Methods

### 2.1. Research Design

This was a prospective, cross-sectional, single-center study.

### 2.2. Participants

The participants were 40 patients with post-stroke upper limb hemiplegia. The inclusion criteria were as follows: age ≥20 years at the time of informed consent, ability to voluntarily move the fingers, and provision of written informed consent prior to participation. The exclusion criteria were: coexisting neurological or psychiatric diseases, an unstable general condition, or other conditions rendering measurement difficult as judged by the raters. 

Sample size was calculated based on the statistical recommendations for intraclass correlation coefficient (ICC) reliability studies by Walter et al. [6]. Setting the minimum acceptable ICC at 0.70, the expected ICC at 0.85, a significance level of α = 0.05 (one-sided), a statistical power of 0.80, and k = 2 measurements per participant, the required sample size was calculated to be 41 participants. The final analyzed sample consisted of 40 participants, one fewer than the prespecified target of 41; therefore, the achieved power was expected to be slightly below the planned 80%.

### 2.3. Criteria for Discontinuation

The study was to be terminated for an individual participant if any of the following events occurred: discovery that the inclusion criteria were not met or exclusion criteria were violated after study initiation; a request by the participant to withdraw; the occurrence of adverse events leading the principal investigator to determine that discontinuation was necessary; or other reasons rendering continuation impossible or inappropriate as determined by the principal investigator.

### 2.4. Study Duration and Location

The study was conducted from August 2022 to July 2024 at Itami Kosei Neurosurgical Hospital, the primary institution of the principal investigator.

### 2.5. Ethical Considerations

Prior to participation, all individuals received a full explanation of the study, and written informed consent was obtained. This study was conducted in accordance with the Declaration of Helsinki (amended at the WMA Fortaleza General Assembly, 2013) and the “Ethical Guidelines for Life Science and Medical Research Involving Human Subjects” issued by the Japanese Ministry of Education, Culture, Sports, Science and Technology; Ministry of Health, Labour and Welfare; and Ministry of Economy, Trade and Industry. The study protocol was approved by the Ethics Committee of Osaka Prefecture University (approval number: 2021-208).

### 2.6. Equipment

**Figure 1:**
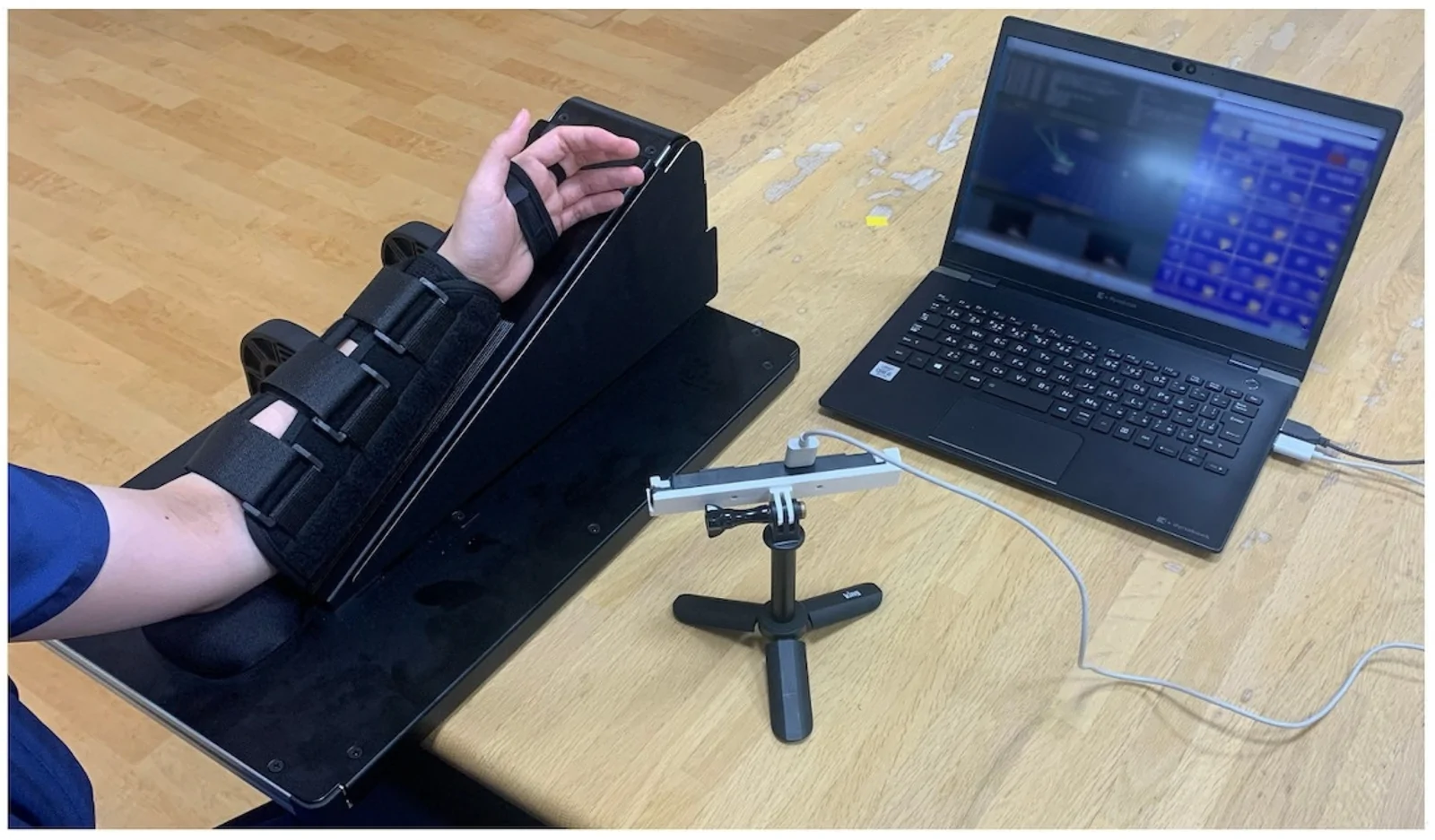
The finger range of motion (ROM) measurement system utilizing the Ultraleap Stereo IR 170 device.

The apparatus used in this study comprised a custom ROM measurement system with in-house measurement software (unversioned, not publicly released; described in [7]) utilizing a Stereo IR 170 camera module (Figure 1), a custom dorsal wrist splint (Apple Medical Co., Ltd., Nagano, Japan), and a manual hand goniometer (SPR-627; Sakai Medical Co., Ltd., Tokyo, Japan).

In the Ultraleap system setup, the camera was positioned laterally to the arm supporter with the subject’s palm facing the sensor, and the forearm was secured using Velcro straps. Before measurements, successful hand tracking was visually confirmed using the reconstructed 3D hand model, with recalibration performed when required. Hand tracking was operated using Ultraleap Hand Tracking Software/Driver v5 and Brekel Hands v1.56, with a nominal sensor frame rate of 90 Hz. The Ultraleap tracking software generated a skeletal hand model by estimating the positions and orientations of the finger bones and joints from the stereo infrared images. The corresponding joint-angle information was derived using Brekel Hands and transferred via Open Sound Control (OSC) to the custom measurement software; no independent joint-angle calculation was performed from the raw optical images. During each complete opening–closing cycle, the maximum flexion and maximum extension values recorded for each joint were extracted as the respective flexion and extension A-ROM values. Further details regarding the design and initial development of the measurement system are described in our previous report [7]. 

### 2.7. Measurement and Evaluation Methods

#### 2.7.1. Participant Demographics and Clinical Characteristics

Demographic and clinical data were extracted from the participants’ medical records. Demographic data included age, sex, and handedness. Stroke-related data comprised the date of stroke onset, side of upper limb hemiplegia, site of brain injury, presence of language comprehension deficits, presence of visual field defects, the Oxford Community Stroke Project (OCSP) classification, and the Brunnstrom Recovery Stage (BRS) for the finger. 

#### 2.7.2. Target Joints

Active Range of Motion (A-ROM) during flexion and extension was measured for all five fingers of the paretic hand. The target joints were the metacarpophalangeal (MCP) and interphalangeal (IP) joints of the thumb, and the MCP and proximal interphalangeal (PIP) joints of the other four fingers.

#### 2.7.3. Measurement Procedure

Ultraleap and manual goniometric A-ROM measurements for each participant were, for almost all participants, completed within the same day. The testing order between the Ultraleap and goniometer measurements was randomized using a random number table. To examine the intra- and inter-rater reliability of the Ultraleap system, one examiner (Rater α) performed the A-ROM measurements twice, and another examiner (Rater β) performed them once on the same participant. To establish the reliability of the goniometric measurements, a third examiner (Rater γ) performed the A-ROM measurements twice using the goniometer on the same participant. Rater γ could be the same individual as Rater α or β. The raters were selected from six occupational therapists (with 3 to 14 years of experience) at Itami Kosei Neurosurgical Hospital. During both Ultraleap and goniometric evaluations, participants were repeatedly instructed to flex and extend their fingers. To ensure consistent movement execution, sufficient rest periods were provided between measurements, and stretching exercises were performed as needed. If significant clinical fatigue was observed, the remaining A-ROM measurement components were suspended and resumed on the following day or two days later (within a strict maximum window of 3 days), after visually verifying the stability of hand motor status. Clinical baseline assessments, including the FMA (specifically the FMA-C subscale), were administered independently within the overall 3-day evaluation window. Additionally, to assess the clinical feasibility of the Ultraleap system, the time required for a single measurement session was recorded for both methods, excluding the equipment setup time.

##### A-ROM Measurement Using Ultraleap

**Figure 2:**
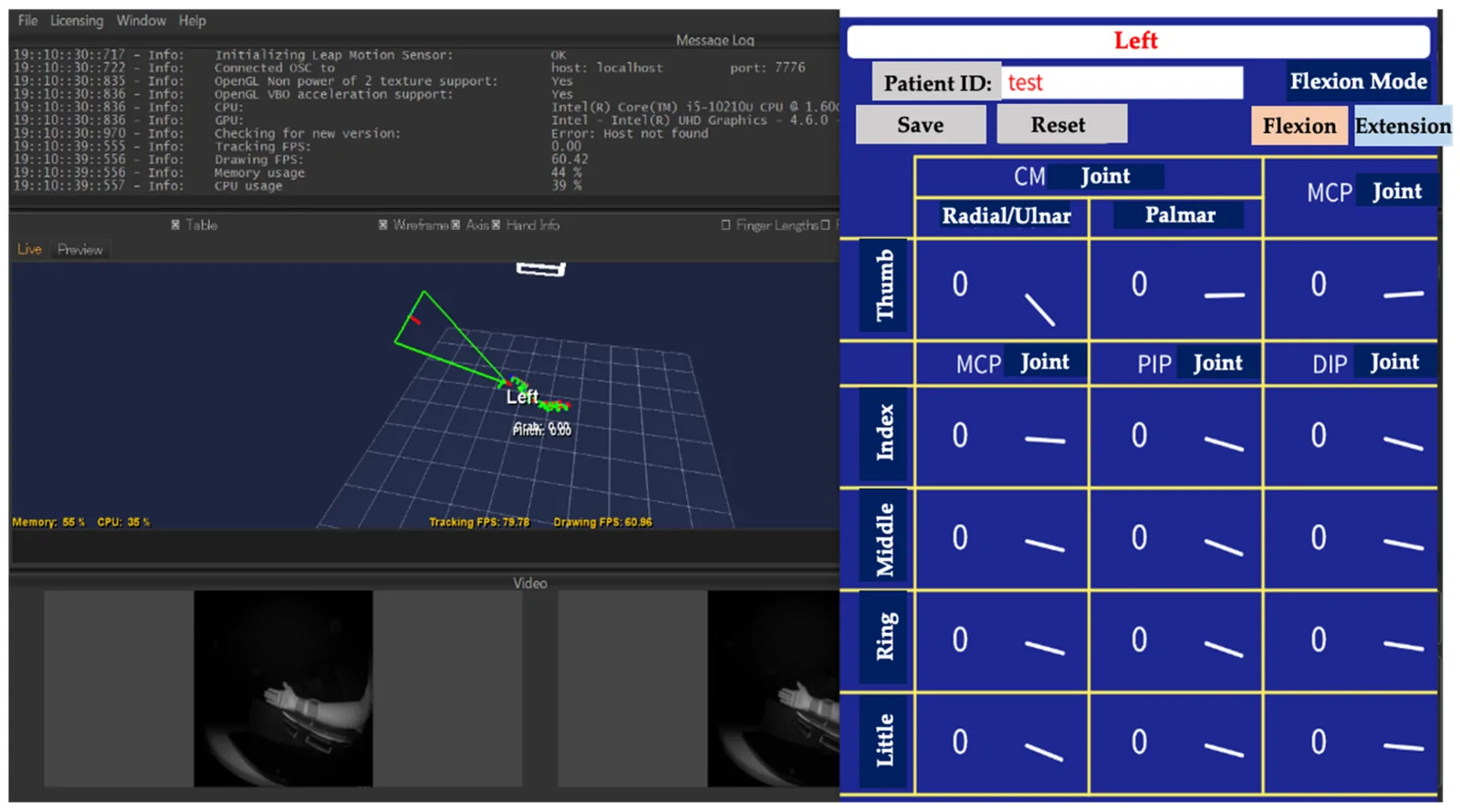
A representative screen capture of the measurement software demonstrating the 3D hand recognition. Although the user interface text is displayed in Japanese, the Ultraleap system automatically renders a digital hand model synchronized in real time with the participant’s paretic hand position.

**Figure 3:**
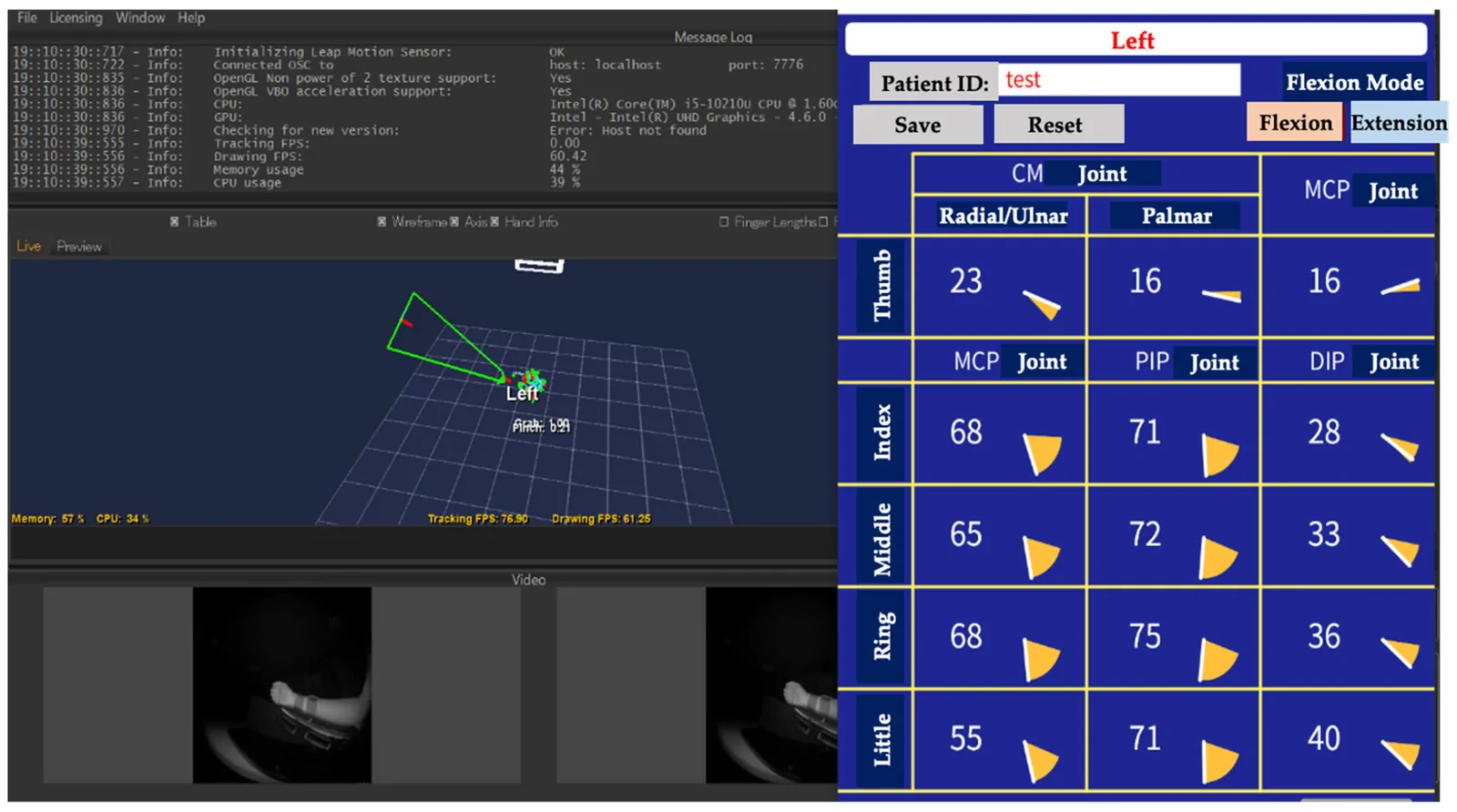
Graphical user interface showing the digital joint angle meters. The virtual meters respond dynamically to the participant’s voluntary finger movements. The software features functional buttons for “Save” (to record joint angles at that specific moment) and “Reset” (to clear the recorded values to zero).

A custom dorsal wrist splint was attached to the participant, and the paretic upper limb was positioned in a neutral forearm posture on the ROM measurement device. The desk height and device position were adjusted to accommodate the participant’s physique. Specifically, the Ultraleap sensor was positioned directly beneath the hand, centered under the middle finger at a vertical distance of approximately 30 cm, oriented upward facing the palmar surface. Minor spatial adjustments in angle and subtle positioning were permitted based on individual physique and optimal hand recognition. The Ultraleap system operated using factory-calibrated settings without requiring additional manual calibration prior to each session, with recalibration performed when required. Once configured, the Ultraleap sensor was oriented toward the palm to recognize the paretic hand, which was then displayed on the monitor (Figure 2). The participant was then instructed to perform finger flexion and extension, and the corresponding joint angles were recorded. The virtual meters for each joint moved dynamically in response to the participant’s voluntary movements (Figure 3). Pressing the save button recorded the joint angles at that specific moment, while the reset button cleared the values to zero. Furthermore, the software allowed for switching between flexion and extension modes during measurement, and joint angles were recorded accordingly.

##### A-ROM Measurement Using a Goniometer

A manual hand goniometer was used to measure the A-ROM of the paretic fingers. Because the goniometric method only allows for the measurement of one joint at a time, making simultaneous evaluation of all joints unfeasible, the measurements were divided into three separate sets for both flexion and extension: (1) the thumb, (2) the index and middle fingers, and (3) the ring and little fingers. Measurements were systematically performed from the thumb to the little finger, and from proximal to distal joints (e.g., thumb MCP, thumb IP, index MCP, and index PIP joints). Furthermore, because it was difficult for participants to maintain the maximum joint angles during the active movement evaluation performed by Rater γ, an assistant was assigned to support the position. To standardize this assistance across participants and raters, all assistants (experienced occupational therapists) were strictly instructed to apply light, static contact solely to maintain the participant’s self-achieved active position, explicitly avoiding any passive overpressure, directional guidance, or joint correction. We carefully ensured that the range of motion during active ROM was not restricted, while providing assistance only at the terminal range. The dorsal alignment method was adopted for all measurements. Prior to the study, the participating occupational therapists underwent extensive training in ROM measurement to ensure methodological consistency.

### 2.8. Statistical Analysis

Statistical analyses were performed using JMP Pro version 18.0.2 and JMP Student Edition version 19.1.2 (SAS Institute Inc., Cary, NC, USA) for macOS, and R version 4.3.2 (R Foundation for Statistical Computing, Vienna, Austria) with the R Commander package version 2.8-0 for Windows. The level of statistical significance was set at 5% (α = 0.05).

#### 2.8.1. Descriptive Statistics and Normality Testing

Descriptive statistics, including means and standard deviations, were calculated for all collected clinical data. The normality of each dataset was evaluated using the Shapiro–Wilk test.

#### 2.8.2. Statistical Analysis for Reliability

To assess intra-rater reliability, the intraclass correlation coefficient (ICC(1,1); one-way random-effects model) was calculated for each joint during flexion and extension across all five fingers. This was based on the first and second measurements obtained by Rater α (for the Ultraleap system) and Rater γ (for the manual goniometer). Inter-rater reliability for the Ultraleap measurements was evaluated by calculating the ICC(2,1) (two-way random-effects model) using the first-run data from Raters α and β. The two-way random-effects model was selected assuming the six raters were representative of a broader population of clinical therapists, allowing generalization of the findings. Reliability coefficients were interpreted according to the criteria established by Landis and Koch [8]. Additionally, Bland–Altman analysis was performed, and Bland–Altman plots were constructed to visually assess the presence of systematic bias (fixed and proportional biases). When a fixed or proportional bias was observed, the limits of agreement (LOA) were calculated to determine the acceptable range of error. Conversely, when no fixed or proportional bias was detected, the minimal detectable change (MDC) was calculated to establish the threshold at which variations between the two measurements could be confidently distinguished from measurement error. The standard error of measurement (SEM) was defined as the standard deviation of the differences (SD_diff_) derived from the Bland–Altman analysis (SEM = SD_diff_/√2), and MDC_95_ was calculated at the 95% confidence level using the formula: MDC_95_ = 1.96 × √2 × SEM ≈ 2.77 × SEM.

#### 2.8.3. Statistical Analysis for Validity

To evaluate criterion-related validity, Pearson or Spearman correlation coefficients were used specifically to assess the concurrent association between the Ultraleap system and manual goniometry, rather than absolute equivalence, based on the normality of data distributions confirmed by the Shapiro–Wilk test. 

In addition, Bland–Altman analysis was performed to assess agreement, systematic bias, and proportional bias between the two measurement methods. For each joint during flexion and extension, the mean difference (systematic bias), standard deviation of the differences, and 95% limits of agreement (LOA; calculated as mean difference ± 1.96 × SD) were determined. Fixed bias was identified by evaluating whether the 95% confidence interval of the mean difference spanned zero and testing whether the mean difference significantly departed from zero using a paired *t*-test (*p* < 0.05). Proportional bias was evaluated using linear regression analysis, in which the difference between the two measurements (Ultraleap–manual goniometer) was regressed on their mean value. Statistical significance for proportional bias was set at *p* < 0.05.

## 3. Results

### 3.1. Participant Flow and Baseline Characteristics

Data were initially collected from 43 participants between August 2022 and July 2024. Of these, three participants were excluded from the final analysis for the following reasons: one withdrew consent, one could not complete the A-ROM measurements due to a technical malfunction of the ROM measurement device and software, and one experienced a decline in mental status during the 3-day evaluation period, making continued participation unfeasible. Consequently, a final sample of 40 participants was analyzed.The baseline demographics and clinical characteristics of the participants are summarized in Table 1. The mean age of the participants was 68.6 ± 13.0 years, comprising 24 males and 16 females. Regarding language impairments, participants with dysarthria or aphasia were included; however, all demonstrated a level of auditory comprehension sufficient to follow verbal instructions, ensuring that their conditions did not interfere with the measurement protocols or study outcomes. No participants exhibited visual field defects. The BRS for the finger was distributed as follows: Stage II (n = 8), Stage III (n = 5), Stage IV (n = 7), Stage V (n = 9), and Stage VI (n = 11). The mean Fugl–Meyer Assessment (FMA-C subscale) score was 8.2 ± 5.5 points. The Shapiro–Wilk test for the FMA-C scores yielded *p* < 0.001, indicating a significant deviation from a normal distribution.

### 3.2. Reliability of Measurements

The intra- and inter-rater reliability coefficients for A-ROM measurements obtained via the Ultraleap system are summarized in Table 2 and Table 3 (intra-rater) and Table 4 and Table 5 (inter-rater). The intra-rater reliability coefficients for manual goniometric measurements are presented in Table 6 and Table 7. For Ultraleap flexion measurements, the intraclass correlation coefficient (ICC) values ranged from 0.576 to 0.896 for intra-rater reliability (Table 2) and from 0.426 to 0.841 for inter-rater reliability (Table 4), indicating joint-specific variability. When examined by anatomical region and joint type, the thumb MCP joint demonstrated an intra-rater ICC of 0.896 and an inter-rater ICC of 0.841, whereas the thumb IP joint yielded an intra-rater ICC of 0.656 and an inter-rater ICC of 0.521. For the MCP joints of the other four fingers, the intra-rater ICCs ranged from 0.792 to 0.893 and inter-rater ICCs from 0.713 to 0.773. In contrast, the PIP joints of these four fingers exhibited lower reliability, with intra-rater ICCs ranging from 0.576 to 0.810 and inter-rater ICCs from 0.426 to 0.655. Overall, flexion measurements revealed lower ICC values at the thumb IP joint than at the MCP joint, and lower ICC values at the PIP joints than at the MCP joints for the other four fingers. For Ultraleap extension measurements, the ICC values for the four fingers (excluding the thumb) ranged from 0.969 to 0.995 for intra-rater reliability (Table 3) and from 0.903 to 0.962 for inter-rater reliability (Table 5). For the thumb, the intra- and inter-rater ICCs at the MCP joint were 0.955 and 0.916, respectively, whereas those at the IP joint were noticeably lower (0.663 for intra-rater and 0.634 for inter-rater reliability). Regarding systematic errors and the MDC, the detailed results are presented in Table 2, Table 3, Table 4 and Table 5. Bland–Altman analysis revealed a significant proportional bias in intra-rater reliability for thumb IP extension (*p* = 0.006) and index PIP extension (*p* = 0.021). For inter-rater reliability, a significant proportional bias was observed for index MCP flexion (*p* = 0.028), while significant fixed biases were detected for index MCP extension (*p* = 0.044) and index PIP extension (*p* = 0.019). The respective limits of agreement (LOAs) for intra-rater reliability were −1.279 to 1.579 for the thumb IP joint (extension) and −3.557 to 0.107 for the index PIP joint (extension). For inter-rater reliability, the LOAs were −1.444 to 3.944 for index MCP flexion, −6.506 to −0.094 for index MCP extension, and −7.712 to −0.738 for index PIP extension.

Regarding the intra-rater reliability of the manual goniometer, the ICC values ranged from 0.871 to 0.934 for flexion and from 0.892 to 0.969 for extension (Table 6 and Table 7).

### 3.3. Validity of Measurements

To examine criterion-related validity in terms of concurrent association, the normality of each dataset was evaluated using the Shapiro–Wilk test. Based on the presence or absence of normality, correlations between the Ultraleap system and the manual goniometer were analyzed using either Pearson’s product-moment correlation coefficient or Spearman’s rank correlation coefficient. During flexion, the correlation coefficients across the joints ranged from −0.071 to 0.493 (Table 8), indicating substantial joint-specific variability. During extension, the correlation coefficient for the thumb IP joint was −0.070, whereas the coefficients for all other joints demonstrated strong positive correlations, ranging from 0.698 to 0.814 (Table 9). 

Furthermore, Bland–Altman analysis revealed systematic and proportional biases between the two measurement methods for both movements (Table 10 and Table 11).

For flexion (Table 10), systematic biases differed between the thumb and finger joints. The Ultraleap system overestimated flexion angles at the thumb joints, with mean differences of 11.33° (Thumb MCP) and 39.45° (Thumb IP). Conversely, it underestimated flexion angles across all four finger joints (Index to Little), with mean differences ranging from −2.90° to −22.30°, showing larger underestimations at the MCP joints. Statistically significant proportional bias (*p* < 0.05) was present across nearly all joints during flexion, indicating that measurement differences expanded with increasing flexion angles.

For extension (Table 11), systematic biases were positive across most finger joints (ranging from 6.45° to 17.40°), indicating mild overestimation by Ultraleap compared to manual goniometry, whereas the thumb showed lower bias (−0.35° for Thumb MCP and −9.40° for Thumb IP). Significant proportional bias (*p* < 0.05) was detected at the thumb IP joint and all MCP joints during extension. In contrast, no significant proportional bias was observed at the PIP joints of the index (*p* = 0.8478), middle (*p* = 0.0816), ring (*p* = 0.2236), and little (*p* = 0.7501) fingers.

### 3.4. Measurement Time

A paired *t*-test demonstrated that the measurement time using the Ultraleap system (49.9 ± 35.2 s) was significantly shorter than that using manual goniometry (396.7 ± 82.5 s; mean difference = 346.7 s, 95% CI [318.3, 375.2]; t(39) = 24.66, *p* < 0.0001). The effect size for this difference was extremely large (Cohen’s d = 3.90).

## 4. Discussion

This study examined the reliability and validity of measuring the active range of motion (A-ROM) of the fingers using a three-dimensional motion capture system (Ultraleap Stereo IR 170) in patients with upper limb hemiplegia following a stroke.

Regarding the assessment of reliability, according to Landis and Koch [8], the following criteria are used to classify intraclass correlation coefficient (ICC) values: 0.00–0.20 as “slight,” 0.21–0.40 as “fair,” 0.41–0.60 as “moderate,” 0.61–0.80 as “substantial,” and 0.81–1.00 as “almost perfect.” Furthermore, Koo and Li [9] point out that ICC values between 0.75 and 0.90 generally indicate good reliability, while values greater than 0.90 indicate excellent reliability. In the present study, the ICC values during flexion exhibited considerable variability in both intra-rater and inter-rater reliability across different joints. For the thumb, reliability was lower at the IP joint than at the MCP joint; for the other four fingers, reliability was lower at the PIP joints than at the MCP joints, indicating that reliability tended to decrease at the distal joints compared to the proximal joints. 

These findings may be attributable to the tracking range within which the Ultraleap system recognizes the fingers. During flexion, when the fingers are clenched, the distal joints can become obscured or occluded from the sensor’s line of sight. Therefore, depending on the angle of the Ultraleap sensor relative to the hand, the system may not have been able to accurately capture the joint axes of the IP or PIP joints. 

Furthermore, the lower ICC values observed for inter-rater reliability compared to intra-rater reliability are thought to be influenced by subject fatigue. Regarding the measurement procedure, since Rater α performed two measurements followed by Rater β performing one measurement, it may have been difficult to maintain equivalent measurement accuracy due to the effects of subject fatigue. In the Ultraleap measurements, two raters conducted the assessments on the same subject. However, due to practical considerations in this study, Rater β performed one measurement after Rater α had completed two measurements; consequently, the estimation of inter-rater reliability may have been confounded by order effects and fatigue. 

On the other hand, with the exception of the thumb IP joint, the ICC values during extension were 0.81 or higher for both intra-rater and inter-rater reliability, indicating “almost perfect” reliability. Koo and Li [9] state that since ICC values are point estimates, their corresponding 95% confidence intervals must be carefully presented and evaluated; however, even when considering the lower limit of the 95% confidence interval, the values for joints other than the thumb IP joint remained at 0.81 or higher, demonstrating high and stable reliability. 

The low ICC value observed specifically for the thumb IP joint is thought to be due to the anatomical positioning and mobility of the thumb. It is well known that the thumb performs not only two-dimensional movements via hinge joints like the MCP and IP joints but also complex three-dimensional movements via the carpometacarpal (CM) joint [10], which is a saddle joint. Since the relative positional relationship between the thumb and the other four fingers changes dynamically depending on the direction of movement at the CM joint, this multi-axial mobility is thought to have caused fluctuations in the calculated joint angles of the MCP and IP joints, leading to lower reliability. Furthermore, previous studies [3] have also shown that thumb joint angles measured using Leap Motion differ from actual joint angles, and the results regarding the IP joint in this study were consistent with those findings.

In contrast, the intra-rater reliability of A-ROM measurements using a manual goniometer showed an ICC value of 0.81 or higher for all joints in both flexion and extension, indicating higher reliability than that reported in previous studies [1,11]. This study was conducted exclusively at the Itami Kousei Neurosurgical Hospital, where the first author is affiliated, and measurements were performed by a small group of six occupational therapists who had received thorough training in range of motion measurement prior to the initiation of the study. Furthermore, the use of standardized goniometers and a standardized measurement posture (utilizing a range of motion measurement device) is considered to be one of the key factors contributing to the higher reliability observed compared to previous studies.

Regarding the criterion-related validity between the Ultraleap system and the manual goniometer, the correlation coefficients for flexion ranged from −0.071 to 0.493, indicating substantial variation across joints. Four main factors may account for this finding. First, as mentioned in the reliability analysis, it is possible that during flexion angle measurement with the Ultraleap system, the distal joints shifted medially, preventing accurate joint recognition. Second, there is an inherent difference in the joint axes targeted by the Ultraleap system and the goniometer. Since manual goniometry typically uses the dorsal method, it measures the lateral side of the joint, whereas the Ultraleap system estimates and measures the internal skeletal model of the joint. Therefore, in subjects with prominent joints, structural differences in measurement values may arise between the two methods. Third, although an assistant was assigned to maintain the subject’s maximum flexion/extension angle independently of the examiner during goniometer measurements, the assistant may have applied slight involuntary pressure to the joint during the measurement. Because this study evaluated active ROM (A-ROM) rather than passive ROM (P-ROM), such subtle external influences likely limited measurement accuracy. Finally, the fourth factor is the influence of inconsistencies in the subjects’ active performance and fatigue. Although rest periods and stretching were provided as needed to ensure consistent flexion and extension performance, standardizing active movement posed inherent limitations, particularly for subjects with severe motor paralysis (BRS II–III).

On the other hand, the correlation coefficients for extension ranged from 0.698 to 0.814 (excluding the thumb IP joint), indicating moderate-to-strong positive correlations. The low correlation coefficient observed for the thumb IP joint (−0.070) is thought to be attributable to the unique thumb position and multi-axial mobility mentioned earlier. Based on these results, the study demonstrated acceptable-to-high reliability and moderate-to-strong correlations with manual goniometry for extension measurements in most joints except the thumb IP joint. However, when taking into account the systematic biases revealed by Bland–Altman analyses and the limited validity observed during flexion, the Ultraleap system cannot yet replace manual goniometry as a direct substitute for absolute ROM assessment. 

Furthermore, Bland–Altman analyses revealed significant systematic and proportional biases during flexion, with the system overestimating thumb IP flexion (up to +39.45°) while underestimating MCP flexion (up to −22.30°). These discrepancies, particularly at larger flexion angles, may reflect a combination of optical occlusion, hand self-shadowing, and procedural differences between the two measurement methods, aligning with previous findings on Leap Motion limitations in stroke populations [12]. Conversely, while extension measurements showed a mild baseline overestimation (6.45–17.40°), no proportional bias was detected at the four-finger PIP joints (*p* > 0.05). Taken together, these results indicate that the Ultraleap system cannot replace manual goniometry for absolute ROM measurement; however, it holds potential as a rapid supplementary tool for repeated measurements of extension. 

Beyond relative reliability evaluated by ICCs, the reporting of absolute reliability metrics—specifically SEM and MDC—provides information regarding measurement error. In the present study, intra-rater extension measurements exhibited small to moderate MDC values across most joints (e.g., 6.017–12.775° for several finger joints). These MDC values represent thresholds above which an observed difference is unlikely to be attributable to measurement error alone under the present measurement conditions; however, they should not be interpreted as evidence of responsiveness or clinically meaningful recovery. Conversely, the substantially larger MDC values observed during inter-rater evaluations (frequently exceeding 12–14°) and during flexion tasks indicate greater measurement variability under these conditions. These findings emphasize the importance of standardized measurement procedures. Further longitudinal studies are required to determine the responsiveness of the Ultraleap system to clinical change over time.

The principal strength of this study lies in the fact that, to the best of our knowledge, it is the first to examine the reliability and validity of finger A-ROM measurements using Leap Motion technology specifically in stroke patients, successfully establishing its metric properties during extension (with the exception of the thumb IP joint). According to the BRS [13], which assesses motor recovery in stroke patients with hemiplegia, finger function typically recovers in a sequential pattern from flexion to extension. In the neurorehabilitation of stroke patients, reliable repeated measurements of finger extension are valuable for clinical application and assessment. Furthermore, a comparison of measurement times revealed that the Ultraleap system enabled a drastic reduction in time compared to manual goniometry, yielding results consistent with previous study [3]. In clinical practice, time efficiency is a critical factor; utilizing digital technology to streamline time-consuming evaluative processes can significantly alleviate the clinical burden on therapists [14]. In this study, as a potential clinical implication of this substantial time reduction, the system may help streamline routine clinical workflows, reduce administrative burden on therapists, and minimize patient fatigue by shortening evaluation sessions. However, given the aforementioned procedural disparities—such as unsupported free-space movements—and optical tracking constraints, these workflow efficiencies must be balanced against the need for cautious interpretation in clinical practice.

This study has several limitations. First, the participant cohort exhibited a predominance of mild paralysis (BRS V–VI), warranting caution when generalizing these findings to acute or severe populations. Second, methodological constraints in the study design—such as the fixed rater sequence (which introduced potential fatigue and order effects that may have underestimated true inter-rater agreement) and minor variations in sensor positioning—may have contributed to measurement variability. Third, a procedural disparity existed in movement support: while manual goniometry utilized light manual contact by an assistant, Ultraleap required unsupported free-space movement to prevent line-of-sight occlusion, which may have systematically widened measurement differences. Fourth, manual goniometry served as a practical clinical reference standard rather than an absolute gold standard, meaning that comparison results inherently reflect the measurement variability of conventional goniometry. Future research should implement randomized rater assignments, standardized mounting fixtures, and multi-camera hardware to mitigate these limitations and further validate the system’s clinical utility.

## 5. Conclusions

In conclusion, this study demonstrated that the Ultraleap system exhibits high intra-rater and inter-rater reliability, along with strong correlations with manual goniometry, for active range of motion measurements during finger extension (excluding the thumb IP joint) in stroke patients. Conversely, performance during flexion and at the thumb IP joint showed considerable variability and proportional biases due to optical occlusion and multi-axial joint constraints. While the system cannot be considered a direct replacement for manual goniometry for absolute ROM measurements, and its clinical application requires careful interpretation in light of procedural and optical constraints, it may serve as an efficient supplementary tool for repeated measurements of finger extension. Future longitudinal studies directly assessing responsiveness to clinical change are warranted before establishing its broader utility.

## Figures and Tables

**Table 1:** Baseline demographics and clinical characteristics of the participants (N = 40).

Characteristic	Value
Age (years), Mean ± SD	68.6 ± 13.0
Sex, n (%)	
Male	24 (60%)
Female	16 (40%)
Handedness, n (%)	
Right	40 (100%)
Left	0 (0%)
Time since stroke onset (days), Mean ± SD	43.9 ± 36.2
Paretic side, n (%)	
Right	17 (42.5%)
Left	23 (57.5%)
Lesion location, n (%)	
Corona radiata	13 (32.5%)
Caudate nucleus	0 (0%)
Putamen	10 (25%)
Internal capsule	5 (12.5%)
Thalamus	4 (10%)
Brainstem	4 (10%)
Cortex	4 (10%)
Other	0 (0%)
Brunnstrom Recovery Stage (Finger), n (%)	
II	8 (20%)
III	5 (12.5%)
IV	7 (17.5%)
V	9 (22.5%)
VI	11 (27.5%)

**Table 2:** Intra-rater reliability, systematic biases, and minimal detectable change in Ultraleap A-ROM measurements in flexion.

			ICC	Fixed Bias (*p*-Value, Status)		Proportional Bias (*p*-Value, Status)		SEM [°]	MDC [°]
			[95% CI]						
Flexion	Thumb	MCP	0.896	0.0878	(Absent)	0.142	(Absent)	1.404	3.893
			[0.812–0.943]						
		IP	0.656	0.451	(Absent)	0.975	(Absent)	2.347	6.505
			[0.439–0.802]						
	Index	MCP	0.792	0.486	(Absent)	0.293	(Absent)	5.406	14.984
			[0.642–0.884]						
		PIP	0.733	0.288	(Absent)	0.522	(Absent)	4.571	12.669
			[0.551–0.849]						
	Middle	MCP	0.857	0.252	(Absent)	0.384	(Absent)	4.614	12.789
			[0.747–0.922]						
		PIP	0.576	0.852	(Absent)	0.388	(Absent)	5.351	14.831
			[0.328–0.750]						
	Ring	MCP	0.866	0.352	(Absent)	0.375	(Absent)	4.512	12.507
			[0.762–0.926]						
		PIP	0.725	0.721	(Absent)	0.0687	(Absent)	4.033	11.180
			[0.539–0.844]						
	Little	MCP	0.893	0.825	(Absent)	0.224	(Absent)	3.515	9.743
			[0.808–0.942]						
		PIP	0.810	0.475	(Absent)	0.351	(Absent)	5.271	14.610
			[0.670–0.894]						

**Table 3:** Intra-rater reliability, systematic biases, and minimal detectable change in Ultraleap A-ROM measurements in extension.

			ICC	Fixed Bias (*p*-Value, Status)		Proportional Bias (*p*-Value, Status)		SEM [°]	MDC [°]
			[95% CI]						
Extension	Thumb	MCP	0.955	0.130	(Absent)	0.461	(Absent)	2.171	6.017
			[0.916–0.976]						
		IP	0.663	0.833	(Absent)	0.00580 *	(Present)	3.161	8.761
			[0.448–0.806]						
	Index	MCP	0.969	0.303	(Absent)	0.773	(Absent)	4.609	12.775
			[0.942–0.983]						
		PIP	0.973	0.0643	(Absent)	0.0205 *	(Present)	4.051	11.229
			[0.951–0.986]						
	Middle	MCP	0.988	0.468	(Absent)	0.308	(Absent)	3.207	8.889
			[0.978–0.994]						
		PIP	0.995	0.369	(Absent)	0.367	(Absent)	2.213	6.133
			[0.990–0.997]						
	Ring	MCP	0.990	0.971	(Absent)	0.331	(Absent)	3.019	8.369
			[0.982–0.995]						
		PIP	0.993	0.178	(Absent)	0.650	(Absent)	2.443	6.772
			[0.987–0.996]						
	Little	MCP	0.985	0.246	(Absent)	0.899	(Absent)	3.224	8.938
			[0.972–0.992]						
		PIP	0.990	0.0999	(Absent)	0.737	(Absent)	2.720	7.538
			[0.981–0.995]						

* *p* < 0.05.

**Table 4:** Inter-rater reliability and systematic biases of Ultraleap A-ROM measurements in flexion.

			ICC	Fixed Bias (*p*-Value, Status)		Proportional Bias (*p*-Value, Status)		SEM [°]	MDC [°]
			[95% CI]						
Flexion	Thumb	MCP	0.841	0.304	(Absent)	0.651	(Absent)	1.715	4.755
			[0.720–0.912]						
		IP	0.521	0.503	(Absent)	0.336	(Absent)	2.978	8.256
			[0.254–0.715]						
	Index	MCP	0.713	0.354	(Absent)	0.0282 *	(Present)	5.957	16.511
			[0.521–0.837]						
		PIP	0.575	0.276	(Absent)	0.794	(Absent)	5.866	16.260
			[0.327–0.749]						
	Middle	MCP	0.773	0.141	(Absent)	0.376	(Absent)	5.723	6.703
			[0.611–0.873]						
		PIP	0.426	0.960	(Absent)	0.755	(Absent)	6.703	18.581
			[0.131–0.650]						
	Ring	MCP	0.728	0.160	(Absent)	0.214	(Absent)	6.170	17.101
			[0.544–0.846]						
		PIP	0.564	0.551	(Absent)	0.667	(Absent)	5.385	14.927
			[0.309–0.743]						
	Little	MCP	0.741	0.477	(Absent)	0.160	(Absent)	5.291	14.665
			[0.561–0.854]						
		PIP	0.655	0.382	(Absent)	0.0754	(Absent)	6.700	18.559
			[0.436–0.801]						

* *p* < 0.05.

**Table 5:** Inter-rater reliability and systematic biases of Ultraleap A-ROM measurements in extension.

			ICC	Fixed Bias (*p*-Value, Status)		Proportional Bias (*p*-Value, Status)		SEM [°]	MDC [°]
			[95% CI]						
Extension		MCP	0.916	0.151	(Absent)	0.900	(Absent)	2.900	8.038
			[0.847–0.955]						
		IP	0.634	0.913	(Absent)	0.0826	(Absent)	3.053	8.461
			[0.404–0.789]						
	Index	MCP	0.921	0.0439 *	(Present)	0.886	(Absent)	7.088	19.647
			[0.851–0.958]						
		PIP	0.903	0.0189 *	(Present)	0.0715	(Absent)	7.710	21.372
			[0.812–0.950]						
	Middle	MCP	0.962	0.094	(Absent)	0.787	(Absent)	5.668	15.711
			[0.923–0.980]						
		PIP	0.942	0.210	(Absent)	0.501	(Absent)	7.195	19.943
			[0.894–0.969]						
	Ring	MCP	0.957	0.391	(Absent)	0.959	(Absent)	6.317	17.511
			[0.921–0.977]						
		PIP	0.916	0.250	(Absent)	0.542	(Absent)	8.707	24.135
			[0.848–0.955]						
	Little	MCP	0.954	0.837	(Absent)	0.215	(Absent)	5.950	16.493
			[0.915–0.975]						
		PIP	0.924	0.119	(Absent)	0.438	(Absent)	7.581	21.013
			[0.861–0.959]						

* *p* < 0.05.

**Table 6:** Intra-rater reliability of manual goniometric A-ROM measurements in flexion.

			ICC
			[95% CI]
Flexion	Thumb	MCP	0.878
			[0.782–0.933]
		IP	0.871
			[0.770–0.929]
	Index	MCP	0.892
			[0.806–0.941]
		PIP	0.919
			[0.853–0.956]
	Middle	MCP	0.917
			[0.849–0.955]
		PIP	0.920
			[0.855–0.957]
	Ring	MCP	0.903
			[0.825–0.947]
		PIP	0.934
			[0.880–0.965]
	Little	MCP	0.882
			[0.789–0.935]
		PIP	0.899
			[0.819–0.945]

**Table 7:** Intra-rater reliability of manual goniometric A-ROM measurements in extension.

			ICC
			[95% CI]
Extension	Thumb	MCP	0.935
			[0.881–0.965]
		IP	0.935
			[0.881–0.965]
	Index	MCP	0.914
			[0.845–0.954]
		PIP	0.951
			[0.911–0.974]
	Middle	MCP	0.920
			[0.855–0.957]
		PIP	0.961
			[0.929–0.979]
	Ring	MCP	0.912
			[0.841–0.952]
		PIP	0.969
			[0.943–0.984]
	Little	MCP	0.892
			[0.807–0.941]
		PIP	0.929
			[0.870–0.962]

**Table 8:** Criterion-related validity of Ultraleap A-ROM measurements compared with manual goniometry in flexion.

			Correlation Coefficient	*p*-Value
Flexion	Thumb	MCP	0.272	0.0892
		IP	−0.0706	0.665
	Index	MCP	0.227	0.160
		PIP	0.493	0.00120
	Middle	MCP	0.387	0.0137
		PIP	0.479	0.00180
	Ring	MCP	0.411	0.00840
		PIP	0.489	0.00140
	Little	MCP	0.446	0.00390
		PIP	0.452	0.00340

**Table 9:** Criterion-related validity of Ultraleap A-ROM measurements compared with manual goniometry in extension.

			Correlation Coefficient	*p*-Value
Extension	Thumb	MCP	0.769	<0.0001
		IP	−0.0700	0.668
	Index	MCP	0.713	<0.0001
		PIP	0.727	<0.0001
	Middle	MCP	0.793	<0.0001
		PIP	0.783	<0.0001
	Ring	MCP	0.814	<0.0001
		PIP	0.742	<0.0001
	Little	MCP	0.806	<0.0001
		PIP	0.698	<0.0001

**Table 10:** Bland–Altman analysis parameters for flexion between Ultraleap and manual goniometry.

			Mean Difference (Bias) [°]	SD of Difference [°]	95% Limits of Agreement (LOA) [°]	Proportional Bias (*p*-Value)
Flexion	Thumb	MCP	11.33	12.75	[−13.67, 36.32]	<0.0001
		IP	39.45	18.56	[3.08, 75.82]	<0.0001
	Index	MCP	−14.18	19.19	[−51.78, 23.43]	0.0264
		PIP	−2.90	12.74	[−27.86, 22.06]	0.0004
	Middle	MCP	−22.30	18.51	[−58.57, 13.97]	0.0068
		PIP	−8.25	13.36	[−34.43, 17.93]	<0.0001
	Ring	MCP	−21.48	16.69	[−54.18, 11.23]	0.0069
		PIP	−10.88	14.26	[−38.82, 17.07]	<0.0001
	Little	MCP	−19.48	21.99	[−62.58, 23.63]	<0.0001
		PIP	−10.23	15.87	[−41.33, 20.88]	0.0244

**Table 11:** Bland–Altman analysis parameters for extension between Ultraleap and manual goniometry.

			Mean Difference (Bias) [°]	SD of Difference [°]	95% Limits of Agreement (LOA) [°]	Proportional Bias (*p*-Value)
Extension	Thumb	MCP	−0.35	9.71	[−19.37, 18.67]	0.0004
		IP	−9.40	21.04	[−50.64, 31.84]	<0.0001
	Index	MCP	6.45	15.83	[−24.58, 37.48]	<0.0001
		PIP	7.75	15.56	[−22.75, 38.25]	0.8478
	Middle	MCP	9.35	16.83	[−23.64, 42.34]	<0.0001
		PIP	11.40	15.68	[−19.33, 42.13]	0.0816
	Ring	MCP	11.50	19.33	[−26.38, 49.38]	<0.0001
		PIP	11.55	16.19	[−20.19, 43.29]	0.2236
	Little	MCP	17.40	17.68	[−17.24, 52.04]	<0.0001
		PIP	9.30	16.20	[−22.46, 41.06]	0.7501

## Data Availability

The data presented in this study are available on reasonable request from the corresponding author. The data are not publicly available due to privacy and ethical restrictions regarding patient information.

## References

[1] Van Kooij Y.E., Fink A., Nijhuis-van der Sanden M.W., Speksnijder C.M. (2017). The reliability and measurement error of protractor-based goniometry of the fingers: A systematic review. J. Hand Ther..

[2] Trejo R.L., Gonzalez Ramirez M.L., Vizcarra Corral L.E., Marquez I.R. Hand goniometric measurements using Leap Motion. Proceedings of the 2017 14th IEEE Annual Consumer Communications & Networking Conference (CCNC).

[3] Nizamis K., Rijken N.H.M., Mendes A., Janssen M.M.H.P., Bergsma A., Koopman B.F.J.M. (2018). A novel setup and protocol to measure the range of motion of the wrist and the hand. Sensors.

[4] Lam W.W.T., Tang Y.M., Fong K.N.K. (2023). A systematic review of the applications of markerless motion capture (MMC) technology for clinical measurement in rehabilitation. J. NeuroEng. Rehabil..

[5] O’Brien M.K., Shin R.Y., Chen C.W., Zhang Y., Jayaraman C., Mummidisetty C.K., Ghaffari R., Rogers J.A., Jayaraman A. (2024). Accuracy of Video-Based Hand Tracking for People With Upper-Body Disabilities. IEEE Trans. Neural Syst. Rehabil. Eng..

[6] Walter S.D., Eliasziw M., Donner A. (1998). Sample size and optimal designs for reliability studies. Stat. Med..

[7] Bandara D.S.V., Arata J. Active range of motion measurement system using an optical sensor to evaluate hand functions. Proceedings of the 2023 45th Annual International Conference of the IEEE Engineering in Medicine & Biology Society (EMBC).

[8] Landis J.R., Koch G.G. (1977). The measurement of observer agreement for categorical data. Biometrics.

[9] Koo T.K., Li M.Y. (2016). A guideline of selecting and reporting intraclass correlation coefficients for reliability research. J. Chiropr. Med..

[10] Neumann D.A. (2016). Kinesiology of the Musculoskeletal System: Foundations for Rehabilitation.

[11] Macionis V. (2013). Reliability of the standard goniometry and diagrammatic recording of finger joint angles: A comparative study with healthy subjects and non-professional raters. BMC Musculoskelet. Disord..

[12] Aguilera-Rubio Á., Alguacil-Diego I.M., Mallo-López A., Cuesta-Gómez A. (2022). Use of the Leap Motion Controller^®^ system in the rehabilitation of the upper limb in stroke: A systematic review. J. Stroke Cerebrovasc. Dis..

[13] Brunnstrom S. (1966). Motor Testing Procedures in Hemiplegia: Based on Sequential Recovery Stages. Phys. Ther..

[14] Levanon Y. (2013). The advantages and disadvantages of using high technology in hand rehabilitation. J. Hand Ther..

